# The Method for Assigning Priority Levels (MAPLe): A new decision-support system for allocating home care resources

**DOI:** 10.1186/1741-7015-6-9

**Published:** 2008-03-26

**Authors:** John P Hirdes, Jeff W Poss, Nancy Curtin-Telegdi

**Affiliations:** 1 Department of Health Studies and Gerontology, University of Waterloo, University Avenue West, Waterloo, Ontario, N2L 3G1, Canada; 2 Homewood Research Institute, Delhi Street, Guelph, Ontario, N1E 6K9, Canada

## Abstract

**Background:**

Home care plays a vital role in many health care systems, but there is evidence that appropriate targeting strategies must be used to allocate limited home care resources effectively. The aim of the present study was to develop and validate a methodology for prioritizing access to community and facility-based services for home care clients.

**Methods:**

Canadian and international data based on the Resident Assessment Instrument – Home Care (RAI-HC) were analyzed to identify predictors for nursing home placement, caregiver distress and for being rated as requiring alternative placement to improve outlook.

**Results:**

The Method for Assigning Priority Levels (MAPLe) algorithm was a strong predictor of all three outcomes in the derivation sample. The algorithm was validated with additional data from five other countries, three other provinces, and an Ontario sample obtained after the use of the RAI-HC was mandated.

**Conclusion:**

The MAPLe algorithm provides a psychometrically sound decision-support tool that may be used to inform choices related to allocation of home care resources and prioritization of clients needing community or facility-based services.

## Background

In Canada, the United States, and internationally, home care is playing an increasingly prominent role in the health care system [1-5], with the aim of reducing costs related to other services, including acute hospitalization and nursing home placement. For example, the Commission on the Future of Home Care in Canada [6] recommended the extension of public funding to cover the cost of four types of home care: post-acute medical care, post-acute rehabilitation, community-based palliative care, and mental health services for clients with behavior-management needs. Although home care accounts only for a small fraction of total expenditures in the Canadian and US health care systems, it has been the sector with the most rapid relative rate of growth in costs [7,8]. In the United States, state 'waiver' programs were introduced with the aim of reducing nursing home utilization among the frail elderly [9]. For example, Michigan's MI-Choice program uses a targeting approach to identify and intervene with those elderly persons at greatest risk of imminent institutionalization in order to prolong their stay in the community.

Despite the widespread belief that home care has the potential to reduce expenditure in other parts of the health care system, there is evidence to suggest that targeted home care probably holds the greatest promise for cost-effective service delivery [10-15]. For instance, if services are provided to a predominantly healthy population, it may be difficult to show any benefits related to home care given that the clients are relatively independent and are at low risk of transition to poor health [16]. On the other hand, if services are provided too late in the disablement process, it may be difficult to reduce the risk of institutionalization [17,18]. The relevant risk factors may be less modifiable and/or the informal network may no longer be able or willing to continue given the level and duration of burden they have endured. Moreover, for persons who are most severely functionally and cognitively impaired, home care may not be a sustainable means of prolonged service delivery due to the demands on formal and informal resources.

Home care case managers are charged with making decisions about the allocation of community and institutional resources for the care of the frail elderly in the community. Their decisions are commonly made on the basis of comprehensive assessments; however, in many jurisdictions these assessments are not standardized, have not been tested for reliability and validity, and are not accompanied by decision-support algorithms that aid in interpretation of the assessment results. Therefore, one might argue that, despite the use of geriatric assessment instruments, the decisions of case managers are often subjectively based and inconsistent due to idiosyncratic differences among clinicians. Clinicians themselves are also often inconsistent over time. A consequence of this type of approach is that limited resources may be allocated ineffectively. There may be a mismatch between needs that are either undetected or discounted inappropriately and the services offered to home care clients. The cost of health care can be driven up by poor communication among health professionals, exacerbations of illness or disability, unnecessary admission to long-term care settings, and avoidable hospitalizations. It is particularly important to provide effective home care to persons at imminent risk of nursing home placement because, once admitted, the likelihood of return to the community declines substantially and the formal costs of care grow substantially.

In 2003, the province of Ontario began to open numerous long-term care facilities across the province. Approximately 15,000 new beds were added to a system of about 50,000 beds in response to perceptions that persons requiring long-term care were using excessive amounts of acute hospital resources. With the opening of these new facilities, the provincial government recognized that there was a need to employ a systematic method of admitting new residents into those beds in order to ensure that the available spaces were taken by persons with the highest level of need and to minimize the chance of premature institutionalization.

In 2002, Ontario mandated the implementation of the Resident Assessment Instrument – Home Care (RAI-HC) [3,4,19,20] for use with all long-stay home care clients in the province. The implementation of the RAI-HC provided a new information source upon which decisions regarding nursing home placements and allocation of community-based services could be made. A review of the literature and consultation with the field was undertaken in order to identify decision-support systems that may aid in the allocation of community and institutional resources. Three main candidate methodologies were identified. First, Miller [21] developed a crosswalk from the Ontario eligibility criteria for long-term care to the RAI-HC. However, this study showed that, using the existing algorithm, approximately 80% of Ontario long-stay home care clients would be eligible for long-term care placement, making this algorithm relatively uninformative.

The INST-RISK system developed by Morris et al [22] is an indexing system that considers approximately 20 functional, medical, social, and psychological measures to develop a summary score of risk of institutionalization, which is then stratified into four risk levels. The Regina Risk Indicator Tool [23] appears to be a derivative of this earlier work in that it makes only minor modifications to the system previously developed by Morris et al. There are a number of limitations with the INST-RISK approach, including: (a) the use of a number of independent variables (such as gender, income) that appear to have little or no predictive power for institutionalization; (b) the application of identical weights to variables of differing levels of importance for institutionalization; and (c) compatibility concerns due to measurement inconsistencies between the INST-RISK items and the RAI-HC.

The third system considered was the MI-Choice algorithm [9,24], which was designed specifically for use with the RAI-HC. MI-Choice was developed for the Michigan Waiver Program using a sample of elderly persons considered to be at imminent risk of institutionalization. Clinical vignettes were used to train case managers on state eligibility rules to standardize their approach to matching clinical need to corresponding community or institutional services. They completed RAI-HC assessments on about 800 home care clients and rated them according to the level of care they believed the client required, including information and referral, homemaking, personal care, professional home care services, and nursing home placement. RAI-HC items were then used to model case manager decisions, beginning with those identified as eligible for nursing home places and information and referral (that is, the extreme values) and ending with homemaking as a residual category. Fries and James [9] reported moderate levels of overall agreement (weighted kappa = 0.55) between the case managers' ratings and the levels of care suggested by the MI-Choice algorithm, independent of the availability of informal support (see [25] for a discussion of kappa values). In the MI-Choice waiver program, this algorithm identified approximately 8% of clients as eligible for nursing home placement; however, in preliminary analyses performed as part of the present study, it classified less than 2% of Ontario home care clients in that category. Therefore, in contrast to Ontario's existing eligibility criteria, the MI-Choice system would allow for very few clients to be considered for entry into long-term care homes. A second concern was that the algorithm appeared to be insensitive to clinical complexity among younger home care clients, who were not part of the original MI-Choice study.

Since no existing system was found to be appropriate for the Ontario context, an effort was launched to develop a new decision-support algorithm for allocating home care resources based on the RAI-HC. The Method for Assigning Priority Levels (MAPLe) was created to assist case managers in determining the relative priority that should be attached to a client regardless of whether he or she needs community or institutional services. Instead of attempting to match client care characteristics to specific venues or types of care, the MAPLe system prioritizes clients to identify those in most urgent need of services, irrespective of the care setting. The aim of this paper is to describe the development and validation efforts undertaken as part of the development of the MAPLe system.

## Methods

### Sample

The study sample for the derivation of the MAPLe algorithm comprised 4,836 clients assessed as part of normal clinical practice by 14 Ontario Community Care Access Centres (CCACs) that implemented the RAI-HC on a pilot basis prior to its mandate by the provincial government. These assessments were completed between 1999 and 2001. Personal identifiers, such as name and health card number, were stripped from the record or encrypted in a way that would prevent identification prior to the transmission of the data to the research group. All assessments were performed by case managers (typically nurses or social workers) and data quality was checked by reviewing the first 10 assessments performed by each newly trained case manager. In addition, statistical checks were made for problems such as missing values and item non-response. Ethics clearance for secondary analysis of the data was obtained through the University of Waterloo Office of Human Research.

The samples for the validation of the MAPLe were obtained through data that were made available to interRAI (developers of the RAI-HC) through license agreements with researchers, service providers, or governments using the RAI-HC. Validation samples included RAI-HC data on home care clients in: Winnipeg Regional Health Authority (WRHA) of Manitoba (*n *= 7,915), British Columbia (*n *= 1,081), Nova Scotia (*n *= 180), Georgia (*n *= 12,761), Michigan (*n *= 19,491), Italy (*n *= 6,151), Iceland (*n *= 297), Sweden (*n *= 178), and Japan (*n *= 3,106). In all settings, the RAI-HC was completed by home care professionals; however, as will be seen later, these different jurisdictions target different populations for home care services. Consequently, these samples differ noticeably with respect to clinical characteristics, such as functional status.

An additional validation sample was available using 2003–2005 RAI-HC data for eight CCACs after the mandated implementation of the RAI-HC began. These data were linked to actual nursing home admissions following the assessment (*n *= 27,234).

### Measures

Data for the derivation of the MAPLe system were almost entirely based on elements available in the RAI-HC assessment. The RAI-HC was created by interRAI [26], a 29-country, not-for-profit research network devoted to conducting cross-national research through the development and application of comprehensive assessment systems for various sectors of the health care system. A number of studies have been completed previously to demonstrate the reliability and validity of the RAI-HC [19,22,27,28] and the instrument is now being implemented in eight Canadian provinces/territories [29] and 15 US states [30]. It was also recently used in an 11-country study funded by the European Union to evaluate home care services in Europe [31]. The RAI-HC comprises an assessment form with approximately 300 clinical elements covering medical, functional, psychological, social, and environmental strengths, preferences and needs of home care clients, a variety of embedded scales that can be used for outcome measurement, and 30 care-planning protocols identifying areas of current or imminent need [19,32-34].

### Analysis

Among the most important methodological and conceptual decisions in this study was the identification of dependent variables to be modeled for derivation of MAPLe. Although the actual institutionalization rate provides a meaningful outcome, the large majority of home care clients do not become nursing home residents in the short or medium term. Therefore, it is appropriate to identify other adverse outcomes that are interim steps along a continuum that may ultimately result in nursing home placement. With this in mind, two other RAI-HC items were identified as dependent variables for this analysis. First, caregiver distress was used, because the collapse of the caregiving network could represent a major risk factor and precursor to institutionalization [35]. Second, the RAI-HC item 'client better off elsewhere' was used to identify the subset of clients who personally felt that they would be, or were rated by others (that is, family or clinicians) as being, better off living in an environment other than the current setting in the community. Hence, there were three binary dependent variables used for this analysis: (i) presence of signs of caregiver distress in the RAI-HC assessment; (ii) rating oneself or being rated by others as being better off elsewhere in the RAI-HC assessment; and (iii) actual nursing home admissions within the next quarter after the RAI-HC assessment. Each of these variables might be considered to be indirect markers of 'need' for additional services in the sense that they are potentially avoidable negative outcomes whose likelihood of occurring may be reduced by high-quality home care services.

The next step was the identification of potential independent variables to be included in the analysis. Candidate items were chosen based on three information sources. First, a detailed review of the literature was used to develop a list of identified risk factors. Gaugler et al [36] recently completed a meta-analysis of the epidemiological literature on nursing home placement, which provides a useful summary of the types of variables that were considered here. Second, items used in the Ontario eligibility criteria crosswalk and in the MI-Choice system were considered. Third, an expert panel focus group meeting was held with clinicians, policymakers, and service providers in home care to identify factors they considered important contributors to risk of institutionalization and adverse outcomes among home care clients. Among the analytic considerations affecting variable selection were: the ability to explain variation in the three dependent variables; avoidance of provider or service variables in favor of person-specific clinical variables; psychometric properties of the individual items; and the risk of misrepresentation of item scores by family members or case managers to increase (or reduce) the client's probability of access to services.

The analysis of the data involved four major steps. First, bivariate analyses were performed to identify individual independent variables that were associated with each of the three dependent variables. This information proved useful in future stages of the analysis for testing alternative models and for identifying candidate items for the decision-tree analysis. Second, decision-tree analysis was performed using SAS Enterprise Miner. This data-mining program uses a combination of automated interaction detection (AID) and computer-assisted regression trees (CARTs), which can be used with continuous and binary independent variables, respectively. The decision-tree analysis began with modeling the item that the client would be better off elsewhere. The criterion for splitting branches of the decision tree was the Gini coefficient, which provides a statistical test of the degree of homogeneity in the groups in different branches. Although Enterprise Miner automatically recommends splits for independent variables, it is possible to override the suggested splits in order to use more clinically meaningful categorizations where appropriate. Therefore, the development of the decision tree was guided by both empirical and clinical choices.

A variety of alternative decision-tree models were presented to a steering subcommittee of the MAPLe development group in order to evaluate the clinical appropriateness and policy implications of different models. This subcommittee played a critical role in identification of the final model. For example, one candidate decision tree split the population according to whether the client lived alone or lived with others as its first split. However, following an extensive discussion by the steering subcommittee, it was recognized that the use of living arrangements as an independent variable in the MAPLe algorithm would result in a problematic steering effect. Family members may become reluctant to take relatives into their homes as frailty worsens for fear that such an action could reduce a client's access to nursing home services, should they be needed. For this reason, living arrangement was excluded as an independent variable from all subsequent decision-tree analyses.

The next stage was to simplify the final decision-tree algorithm by combining groups with relatively homogeneous levels of risk. For example, 32–34% of clients who had different combinations of ADL impairments, cognitive impairment, and behavior disturbances were considered better off elsewhere. Those groups could be combined to form a single very-high-risk group, based on their similar risk profiles. This refinement of the decision tree for the better off elsewhere item resulted in five hierarchical levels ranging from low risk to very high risk. This new, simplified algorithm was then applied to the other two dependent variables: actual nursing home admissions within the next quarter and caregiver stress. Logistic regression models were used for the three dependent variables using the interim MAPLe algorithm in order to identify inconsistencies in risk levels between the three dependent variables. Modest adjustments were made in order to correct for some inconsistencies between the model results, such that the final algorithm yielded a logistic regression model with roughly equivalent increments in risk levels with each increase in the MAPLe score. The final step was to validate the MAPLe algorithm against the other Canadian, US, and international data to determine the extent to which MAPLe yielded similar results across provinces and cross-nationally.

## Results

**Figure 1:**
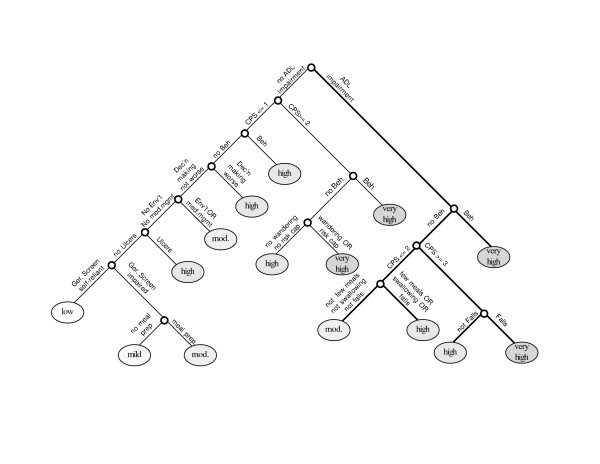
Schematic representation of the MAPLe algorithm.

Figure 1 provides a schematic representation of the final MAPLe algorithm [37]. The measures used to differentiate the level of risk for all three outcomes of interest were: ADL impairment, cognitive impairment, behavior disturbance, decline in decision-making, problems with medication management, pressure ulcers or stasis ulcers, environmental challenges, falls, inadequate meals, problems with meal preparation, difficulty swallowing, and the RAI-HC's nursing home risk care-planning protocol. In the derivation sample, this algorithm yielded five different groups ranging from low to very high risk. The low-, mild-, and moderate-risk categories contain approximately one-quarter of the sample each. The high-risk category included 18% and the very-high-risk group was about 7% of the sample.

Table 1 provides the distribution of MAPLe priority levels in 10 different jurisdictions. Iceland, Manitoba, Sweden, and Ontario were similar in that the majority of home care clients were skewed toward the low, mild, and moderate MAPLe priority levels. In each of these jurisdictions, less than 10% of the clients were in the very high MAPLe levels. In contrast, the Michigan, Georgia, Japan, and Italy samples were strongly skewed toward the moderate, high, and very high MAPLe levels, with less than 10% of clients falling in the low and in the mild MAPLe categories. The distributions of the Nova Scotia and British Columbia samples fell into an intermediate range between these two extremes.

**Table 1:** Distribution of MAPLe priority levels in 10 jurisdictions

	**MAPLe**					
		
**Jurisdiction**	**Low**	**Mild**	**Moderate**	**High**	**Very high**	***n***
Iceland	39.4	12.8	20.9	22.9	4.0	297
Manitoba	32.9	17.5	24.6	20.9	4.1	7,915
Sweden	32.0	12.4	35.4	14.6	5.6	178
Ontario	24.4	22.6	28.1	17.6	7.3	4,836
Nova Scotia	23.3	10.0	31.7	24.4	10.6	180
British Columbia	12.9	13.1	20.8	37.5	15.7	1,081
Michigan	5.7	7.2	42.0	31.9	13.2	19,491
Georgia	0.5	1.5	52.4	34.4	11.1	12,761
Japan	5.3	3.6	37.1	33.3	20.8	3,106
Italy	3.5	1.8	33.7	39.6	21.5	6,151

Table 2 provides the Ontario results for the algorithm derivation by comparing MAPLe priority levels against the three dependent variables. With respect to nursing home admissions, there is a substantial increase in risk of admission within the next 90 days for every increment in MAPLe. Compared with the low MAPLe level, the risk of nursing home admission increases threefold for the moderate category and tenfold for the very high MAPLe level. Although these relative changes in risk are substantial, the large majority of clients were not admitted within the next 90 days, including those in the very high MAPLe category. Only 14.5% of those in the very high level were admitted within 90 days; however, the annual risk would be much higher because the increments in placement rates are likely to be cumulative over time. The pattern for caregiver distress is more pronounced than that of nursing home placement. Both the absolute and relative differences across MAPLe categories are greater than the results for nursing home admission alone. There is a fivefold higher risk of caregiver stress comparing the moderate to the low MAPLe categories, and a 26-fold higher risk comparing the very high to the low MAPLe categories. In absolute terms, the difference between the very high and low MAPLe categories is a 47.4% increment in the rate of caregiver distress. Similar patterns are also noted for clients being rated as better off elsewhere. Although the increments in the relative rise in risk of being rated as better off elsewhere are not as large as with the other two dependent variables, the absolute differences between MAPLe groups remain substantial.

**Table 2:** Percentage of clients entering long-term care home, caregiver distress, and ratings of being better off elsewhere by MAPLe priority level, Ontario derivation sample (*n *= 4,835)

	**MAPLe**				
	
**Dependent variable**	**Low**	**Mild**	**Moderate**	**High**	**Very high**
***Admitted to long-term care home within 90 days***					
Absolute rate	1.3%	2.9%	4.7%	7.5%	14.5%
Adjusted odds ratio*	Reference	2.14	3.64	5.91	11.35

***Caregiver distress***					
Absolute rate	3.6%	8.0%	15.8%	26.1%	51.0%
Adjusted odds ratio*	Reference	2.50	4.96	9.31	26.61

***Better off elsewhere***					
Absolute rate	6.4%	9.1%	10.2%	18.9%	33.1%
Adjusted odds ratio*	Reference	1.40	1.67	3.39	7.10

***n***	1,181	1,094	1,358	852	351

* Adjusted for age, sex

Table 3 provides the cross-jurisdictional results for caregiver distress. Although there were some minor discrepancies within countries between adjacent MAPLe categories, there was a clear trend of increased rates of caregiver distress with higher levels of MAPLe. It is interesting to note, however, that the relative rate of change in caregiver stress across MAPLe levels varies noticeably across cultures. For example, the two Nordic country samples show marked increases in caregiver distress at the very high levels, and a similar pattern is found in British Columbia. These results are also evident in jurisdictions where there is a tendency towards a higher proportion of home care clients in the high MAPLe categories. For example, in both Michigan and Italy, there is a strong linear increase in rates of caregiver distress with MAPLe levels. On the other hand, while the rates still increase with higher MAPLe levels, the trend is relatively flatter in Georgia and Japan.

**Table 3:** Cross-jurisdictional comparison of rates of caregiver distress by MAPLe priority level

	**MAPLe**				
	
**Sample**	**Low**	**Mild**	**Moderate**	**High**	**Very high**
***Derivation sample***					
Ontario	3.6	8.0	15.8	26.1	51.0

***Validation samples***					
Italy	14.5	21.8	28.5	40.2	55.8
Michigan	11.9	12.2	26.1	33.5	52.1
Iceland	6.0	15.8	8.1	16.2	50.0
Sweden	1.8	9.1	14.3	15.4	50.0
British Columbia	8.6	8.5	16.4	36.1	48.8
WRHA, Manitoba	5.7	10.2	15.7	23.5	41.2
Ontario (8 CCAC)	4.2	7.4	14.1	23.3	41.0
Nova Scotia	0.0	5.6	19.3	38.6	36.8
Japan	15.2	5.4	19.7	14.7	18.6
Georgia	3.0	7.8	8.5	13.6	13.1

Table 4 provides the cross-jurisdictional results for ratings that the client would be better off elsewhere. Again, there is a relatively consistent pattern where higher MAPLe levels translate into a higher proportion of clients being rated as being better off elsewhere. The only clear exception to this trend is in Japan, where the MAPLe low category is most likely to be rated as better off elsewhere. However, only about 5% of the Japanese sample fell into the MAPLe low category, and may represent an unusual subpopulation of Japanese home care clients.

**Table 4:** Cross-jurisdictional comparison of percentage of clients rated as better off elsewhere by MAPLe priority level

	**MAPLe**				
	
**Sample**	**Low**	**Mild**	**Moderate**	**High**	**Very high**
***Derivation sample***					
Ontario	6.4	9.1	10.2	18.9	33.1

***Validation samples***					
Italy	4.7	11.8	7.1	9.0	19.0
Michigan	4.2	5.4	6.8	7.9	13.8
Iceland	10.3	26.3	22.3	29.4	50.0
Sweden	3.5	9.1	4.8	11.5	10.0
British Columbia	10.8	14.1	18.7	33.8	47.7
WRHA, Manitoba	5.1	8.4	10.8	16.9	23.5
Ontario (8 CCAC)	6.3	9.2	13.6	25.0	43.0
Nova Scotia	4.8	11.1	28.1	54.6	84.2
Japan	14.6	5.4	7.8	8.2	10.9
Georgia	3.0	5.7	5.4	6.0	7.3

Table 5 provides results for the Ontario and the Manitoba samples on two variables that were not considered in the derivation of MAPLe: formal community service cost and hours of informal care. This analysis is limited to Ontario and Manitoba because these are the only Canadian samples based on large-scale pilot implementations of the RAI-HC, rather than smaller research studies. Therefore, these samples are more likely to reflect the actual pattern of service provision and informal care in the population of home care clients in these two provinces. With respect to formal service costs, higher MAPLe priority levels are associated with higher weekly costs of formal care; however, the trend appears to be somewhat non-linear with a threshold level evident at the MAPLe moderate category. That is, for the low and mild categories, the costs of formal services are approximately $100 CDN per week, compared with between $180 CDN and $300 CDN per week for the three higher MAPLe categories. Informal care follows a somewhat different trend in the sense that there is a more linear relationship between MAPLe levels and hours of informal care estimated by caregivers. The differences are pronounced across MAPLe levels, ranging from 7.3 hours in low MAPLe clients in Ontario to 36.0 hours in their very high counterparts. Therefore, it is not surprising that higher MAPLe levels are also associated with higher levels of caregiver distress.

**Table 5:** Mean weekly formal and informal care by MAPLe priority level, Ontario derivation sample and WRHA Manitoba

	**MAPLe**				
	
**Resource use**	**Low**	**Mild**	**Moderate**	**High**	**Very high**
***Mean weekly cost of formal care ($CDN)***					
Ontario (derivation sample)	85.6	97.2	185.2	194.1	219.7
WRHA, Manitoba	94.9	106.1	227.2	295.9	277.3

***Mean weekly hours of informal care***					
Ontario (derivation sample)	7.3	10.2	21.9	25.4	36.0
WRHA, Manitoba	5.5	8.0	16.1	20.3	30.1

**Figure 2:**
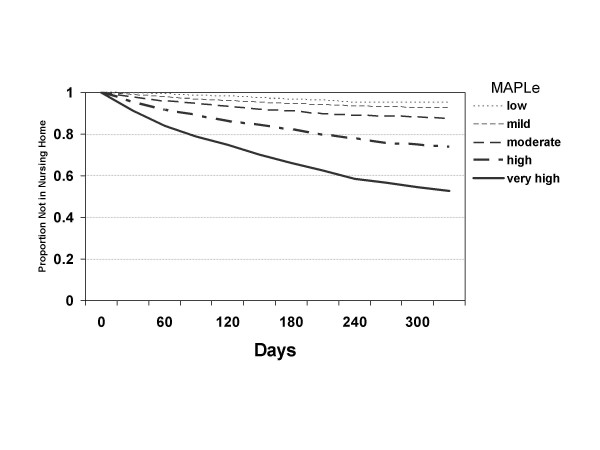
Survival plot of time to nursing home admission by MAPLe priority level, Ontario (eight CCAC validation sample).

Figure 2 shows a survival plot of time to nursing home admission by MAPLe priority level using the eight CCAC validation sample for Ontario. Longitudinal data were not available from the other jurisdictions, so it was not possible to replicate these analyses outside of Ontario. The survival plot shows a clear separation of nursing home admission rates for all five MAPLe levels. After one year of follow up, 4.8% of clients in the MAPLe low level were admitted to nursing homes, compared with 47.4% of those in the very high level.

## Discussion

MAPLe provides an empirically sound decision-support system that will allow case managers to make more systematic evaluations of the needs of clients and the urgency with which they should respond to those needs. Using three different outcomes, with validation results in six countries, MAPLe clearly differentiated the risk of adverse outcomes, including institutionalization. Case managers who have completed an RAI-HC assessment can obtain the MAPLe results automatically from software in which the algorithm is embedded and these results then provide a context against which person-specific service recommendations may be made. It should be noted, however, that the intent is not to use MAPLe as an automated decision-making system devoid of clinical judgment. Instead, case managers considering MAPLe scores should also engage in a full discussion with the client, family, and other formal service providers to develop person-specific recommendations that take into account the individual's strengths, preferences, and needs. For example, some clients who score low on the MAPLe algorithm may rate themselves as being in poor health, may be showing signs of depression, or may have had an overnight hospital stay or frequent emergency room visits. It would be inappropriate to treat such an individual as an 'information and referral only' client if the case manager believes these other clinical considerations to be of critical importance. On the other hand, individuals in the very high MAPLe category do not necessarily require immediate nursing home placement because, for example, they may have family members who are both willing and able to address their current level of need in the community. Similarly, alternative housing arrangements may provide these individuals with the appropriate resources to remain relatively independent with access to formal supports when needed.

While MAPLe may be used to make person-centered resource allocation decisions at the individual level, it may also be used as a monitoring system at the regional, organizational, national, and international levels to evaluate practice patterns. That is, one may stratify populations according to MAPLe levels and then compare the performance of home care agencies with respect to outcomes of care within MAPLe levels. Such a benchmarking system may be used to identify jurisdictions where MAPLe-adjusted nursing home admissions, for example, are higher than expected based on the experience of other regions. Similarly, MAPLe levels at intake can be used to examine regional variations in access to services by level of need.

The introduction of MAPLe into normal, daily use in home care can be expected to have an impact on the nature of services provided by agencies. That is, clients with lower MAPLe levels will be less likely to receive services than they may have been in the past, and clients with higher MAPLe levels will presumably be more likely to access those services because they are more readily identifiable. That being said, it is not necessarily true that subgroups of home care clients need to be excluded from all services based on low MAPLe scores. The choice of who receives services and what type of services are provided remains a value-based decision to be made by policymakers and clinicians in each jurisdiction. In that regard, the cross-national results for MAPLe shown in Table 3 do not imply that some countries are providing services correctly while other countries erroneously target light-care clients. The main benefit of implementing MAPLe would be that persons with higher levels of need should be at a relatively higher level of priority for access to services than those with lower-level needs. That does not preclude the possibility of persons at the lowest level receiving appropriate services.

The present study made some important decisions regarding the role of informal support in the development of the prioritization system for community and institutional services. The choice to include measures of the informal support system (that is, caregiver stress) as a dependent variable rather than as an independent variable was important for methodological, clinical, and policy-related reasons. From a methodological perspective, the use of measures of informal support as independent variables would create a situation where those variables become highly vulnerable to systematic response bias in order to gain access to desired services when used in normal clinical practice. That is, if family members believe that saying they could do more to help a client would place their relative in a lower priority level, there would be a strong disincentive to making such claims. Therefore, any items reflecting the capacity of the informal network to provide care could become less valid after the implementation of such a system. The introduction of this type of bias will make it more difficult to differentiate between families who are showing clear signs of distress and those who have continued capacity to provide more services. From a policy perspective, one must be concerned about the potential unintended consequences of a steering effect that would result in family members being less likely to provide informal care. The capacity of the home care system to function adequately is heavily dependent on the provision of informal care by family members. Extreme caution should be taken with the introduction of policy measures that may provide disincentives to families to be involved. At the same time, distress among caregivers should be a concern to home care service providers and policy makers.

Although the present study showed that the MAPLe system is related to both the formal costs of service provision and hours of informal care, MAPLe is not intended to be an alternative to case-mix systems (for example, as an alternative to the RUG-III/HC [38] system). That being said, the introduction of MAPLe will have important implications for case-mix distributions in both home care and in long-term care facilities. As MAPLe begins to be used, one might expect the relative resource intensity of home care clients to increase because greater priority will be given to clients with higher levels of need. Similarly, to the extent that MAPLe is used as a decision-support system for nursing home placement, it is likely to result in a shifting of the distribution of new admissions to long-term care settings towards those community-based clients with the highest levels of need. Therefore, the introduction of MAPLe may have the effect of increasing case-mix or resource intensity in both home care and nursing home settings.

This study also demonstrates the benefits of using a grouping methodology, as with decision-tree analysis, compared with indexing systems that simply assign a linear increase in scores for any combination of variables. Unlike systems such as the RRIT or INST-RISK, MAPLe is able to consider the impact of specific combinations of variables (for example, cognitive impairment combined with functional impairment).

There was a remarkable level of cross-jurisdictional consistency in the MAPLe results. Although the slope of the relationship between the MAPLe scores and the dependent variables of interest were not always identical between jurisdictions, the relative increments in risk between MAPLe levels was very consistent in all of the countries examined. This is particularly surprising given the value differences underlying the provision of community-based services in these jurisdictions. Japan and Italy, for example, tend to be traditional in their expectation that family members will provide the bulk of care. It is therefore not surprising to see lower absolute levels of ratings that clients would be rated by themselves or by others as being better off elsewhere in these jurisdictions, compared with the Canadian results. On the other hand, in all jurisdictions, there was a rather consistent increase in the proportion of clients rated as being better off elsewhere with each increment in the MAPLe level. It would be useful to conduct future research in these other countries to determine the extent to which MAPLe is indeed predictive of actual institutionalization in those settings. In addition, it is important to recognize that clients, family members, and home care professionals may not always share the same view about the most appropriate care setting. These potentially different perspectives should be taken into account when determining what services would be most appropriate for a given client.

An important next step for research with the MAPLe system is to conduct intervention studies to determine the extent to which the level of risk associated with a given MAPLe category can be reduced. For example, could additional measures be put in place that would have the result of yielding a lower than expected institutionalization rate within a MAPLe category, or could the risk factors that have placed the client in that MAPLe category be reversed? For instance, falling is a major factor contributing to higher MAPLe scores. If a falls intervention was put in place that actually prevented future falls, conceivably, the individual's MAPLe scores would decline over time.

It will also be important to conduct research into the extent to which MAPLe is relevant to home care outcomes other than caregiver distress or nursing home placement. For example, it is not necessarily the case that clients with higher MAPLe scores feel more socially isolated or lonely or that they would benefit from interventions such as friendly visiting programs or transportation services. Therefore, it would be helpful to consider what comprises the full spectrum of benefits that could be realized through high-quality home care. This might be done through qualitative studies of home care clients, caregivers, case managers, and administrators. Once these benefits are identified, further research should be conducted on the effectiveness of MAPLe as a targeting system for persons at risk of adverse outcomes in other domain areas.

MAPLe also has potential benefits for the support of cross-national research. The problem that population-level information cannot be equated across jurisdictions is an important limitation of any home care research that is not based on individual-level observations. Indeed, results in Table 1 show that it would be a faulty assumption to believe that home care clients in Italy are comparable with home care clients in Ontario, for example. However, by using the MAPLe system to stratify samples with individual-level data, one could be more confident in the equivalence of samples being compared across two or more jurisdictions.

## Conclusion

The MAPLe algorithm provides an empirically based decision support tool that may be used to inform choices related to the allocation of home care resources and prioritization of clients needing community or facility-based services. MAPLe is a valid predictor of nursing home placements, caregiver distress and ratings that the client would be better off elsewhere, and it has been shown to perform well in a variety of international jurisdictions. MAPLe may be used at the individual level to support clinical decision-making, but it may also be used with aggregated data to inform policy development and planning.

## Competing interests

The author(s) declare that they have no competing interests.

## Authors' contributions

JPH led the study design and data analysis and wrote the draft manuscript. JWP prepared the data, collaborated in data analysis, and co-wrote the manuscript. NCT provided clinical advice to support the data analysis and interpretation of data. The final manuscript was reviewed and approved by all authors.

## Pre-publication history

The pre-publication history for this paper can be accessed here:


